# “That was really a productive segment, wasn’t it?” Nonverbal markers of humor in the American presidential debates of 2016 and 2020

**DOI:** 10.3389/fpsyg.2024.1453168

**Published:** 2025-04-08

**Authors:** Sabina Tabacaru

**Affiliations:** ^1^Département d’Etudes des Pays Anglophones, UFR LLCER-LEA, Université Paris 8, Saint-Denis, France; ^2^UR TransCrit, Saint-Denis, France

**Keywords:** humor, sarcasm, stance, multimodality, nonverbal, political debates

## Abstract

These past few years have marked a growing interest in multimodality, interaction and eye-gaze in the interpretation and understanding of discourse. Eye-gaze, for example, plays a central role in face-to-face interaction and stance taking because it helps discourse participants coordinate with each other. Such visual markers help the interlocutors/audience to intersubjectively connect to the same common ground on which they construe their meanings. The case of humor has also received more attention from a multimodal perspective since it follows the same patterns of meaning construction and coordination. Elements that are salient to the humorous interpretation will be emphasized using either prosodic cues or visual markers, such as facial expressions and head movements. In this paper, we explore the use of such nonverbal discourse markers with the use of humor in the American presidential debates of 2016 and 2020, analyzing their role on the humorous stance.

## Introduction

Humor holds different functions in conversation (Hay, 2000), and it has been a point of interest for linguists, psychologists, anthropologists, among others. As a research path, it provides complex analyses of discourse since speakers need to make their intentions salient to the other participants. As opposed to other types of analyses of discourse, humor relies on the element of incongruity, where meanings overlap, giving rise to different effects that the speakers need to make obvious to their interlocutors. Incongruity thus implies changing between different meanings, or frames, in order to switch the viewpoint on which the interpretation relies. I align here with frameworks of humor in terms of a layered mental space configuration, as has been explained elsewhere (Brône, 2008) in which meaning is formed as a pretense space on top of a discourse space (see also Tabacaru, 2019 or De Vries et al., 2021).

Moreover, humor has been linked to persuasion (Li and Pryor, 2020), particularly in political arguments used for different purposes (Feldman, 2024), which will also be the case here. For example, Li and Pryor (2020) find that, in oral arguments, there is a positive reaction to the people who cause laughter as they win more votes from justices in the Supreme Court. As such, humor would have an effect on the power of persuasion.

The aim of the present paper is to look at semiotic markers of humor in interaction in order to analyze the role they have in (humorous) meaning construction in the specific context of political debates, where participants need to argue in favor of their position, as opposed to the one presented by their adversary. Hence, the viewpoints presented are different, contradictory, and each participant needs to persuade an audience that will cast a vote. The understanding of different viewpoints (Dancygier and Vandelanotte, 2016) is central to this type of discourse in order for interlocutors to make the appropriate responses, be their humorous or not. This echoes the theory of mind (Baron-Cohen, 1995; Tomasello, 1999) because each participant needs to understand their opponent’s position as well as the complex meanings created through implicature (Grice, 1975).

At the heart of the debate, the emphasis falls on the question of stance, which is understood, according to Du Bois (2007: 139) as “the power to assign value to objects of interest, to position social actors with respect to those objects, to calibrate alignment between stancetakers, and to invoke presupposed systems of sociocultural value.” Speakers thus have to position themselves in relation to their opponent and certain topics that are the subject of the debate. It is thus argued that humorous meaning plays a central role in the way stancetakers evaluate their opponent’s discourse, by directly criticizing or by switching the viewpoint to a negative one.

In the following, closer attention is paid to interaction and multimodality in discourse, from the perspective of humorous communication.

## Humor and interaction

While traditional approaches to humor (Raskin, 1985) paid attention to the semantic and/or pragmatic background that helps create jokes, more recent studies emphasize the role interaction plays in these types of exchanges (Priego-Valverde, 2009; Feyaerts et al., 2015; Brône and Oben, 2013; Feyaerts, 2013a; Feyaerts, 2013b; Feyaerts and Oben, 2014; Tabacaru, 2019, among others). From this perspective, both speaker and interlocutor have to be taken into consideration in the way these meanings are created and understood. Such studies focus on questions of intersubjectivity and common ground, which are fundamental in meaning coordination among speakers. A shared common ground (Clark, 1996), for example, allows the speaker to know which processes their interlocutor will go through in order to understand their message as humorous (see, for example, Yus, 2003, who speaks about the search for relevance). Drawing from shared background assumptions, the speakers are able to coordinate with each other’s meanings and behaviors, using the context and discourse at hand. As Geeraerts (2021: 24) puts it: “Ideally, if A and B are paying attention to Z, the common ground is maximal if both A and B believe that the other focuses on Z, and if A and B both believe that the other is aware of the fact that they focus on Z.” The speakers thus make references to the same common ground that *they believe* they share with the others. Implied meanings are thus to be understood against a common ground that discourse participants share with each other and that they enrich in conversation.

In order to illustrate this, consider the following example which comes from the present corpus (Trump versus Biden in 2020) and represents an example of humor in interaction. As the debate and the presidential campaign take place during the Covid-19 pandemic, the moderator talks about regulations regarding crowds, stating that the two candidates have different approaches, i.e., Trump prefers to campaign in large crowds, whereas Biden organizes smaller events. The humor is construed by Trump in his last reply, building on what the moderator said:

**Table:** 

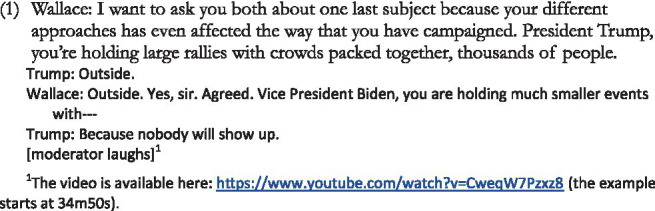

This represents an example of *dialogicality*, which, as defined by Du Bois (2007: 140) is when “a stancetaker’s words derive from, and further engage with, the words of those who have spoken before.” The viewpoint is shifted as, at first, the focus of attention is on the crowds that gather to see the candidates, and the moderator here uses the adjectives *large* and *smaller* to describe each candidate’s campaign. Of course, the larger crowds would mean more risk of contracting Covid-19, but Trump emphasizes that he holds these rallies outside. This implies that he respects the regulations and minimizes the risks taken. When the word *smaller* is used, which highlights the comparison to his *large* rallies, Trump adds a different perspective: he can organize large events because people want to see him whereas Biden cannot organize large events as nobody will attend these. This is also a case of reasoning (if P, then Q), with different implied meanings:

**Table:** 

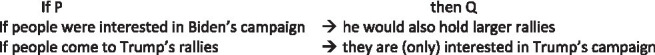

The shift of focus from him to his opponent also shows Trump’s understanding of the different implicatures between *larger* and *smaller crowds* in the context of the pandemic. In order to shift the viewpoint in the given context and reverse the negative implication from him to Biden, he needs “deep cognitive coordination” (Verhagen, 2005: 4) with the other speaker, as well as the audience watching the debate.^1^

As further noted by Verhagen (2005: 7-8), “a speaker […] is committed to the assumption that her utterance is in principle interpretable by someone else sharing the knowledge of certain conventions.” In this case, Trump knows very well the audience can access the same negative implicatures that he did regarding his campaign and the larger crowds attending his rallies. This is made clear by Verhagen (2005: 12): “The inferential load of utterances is crucially involved in the way they relate to each other in connected discourse. Discourse consists of chains of inferential steps, including the possibility of rejecting one or more steps and ‘changing course’.” This is what happens here, as Trump needs to reject the negative implicatures and put the focus on his opponent rather than his own behavior and campaign. Even though the moderator never specifically says that this comparison is negative, this is interpreted as “negative evaluation” (Hunston, 2007: 41) by Trump, who then has to change the viewpoint and the course of the debate.

The use of the modal *will* is linked to certainty, as, from this point of view, there is no doubt that people do not want to attend Biden’s rallies. This fits the category of generalization (see Zhang, 1998, and Scheibman, 2007), which represents a comment on Biden’s entire campaign. From Trump’s point of view, their Covid-19 behaviors are divergent between the two candidates, but for different reasons. This creates humor in the way the implicatures are created as it has to be interpreted against a shared common ground: in this case, the Covid-19 pandemic and the regulations surrounding it. This example, as many others from the corpus, can be seen as sarcastic, which is represented as a pretense (Clark and Gerrig, 1984; Barnden, 2017) with “clearer markers/cues and a clear target” (Attardo, 2000, 795). Even against Grice’s cooperative principle (for example, the maxim of quality), the incongruity between what is real and what is pretense is clear: Trump targets his opponent by creating a pretense space in which people would not attend his rallies because they are not interested in his campaign.

In face-to-face interactions, as is the case here, participants also make their humorous implicatures clear to the interlocutors/audience, through their verbal and non-verbal behavior (see De Vries et al., 2021). Multimodality thus plays a central role in the way speakers coordinate meanings with each other.

## Multimodal markers of humor

There is an increasing focus on the way the body is used in interaction, as a marker of stance (see, for example, Bateman et al., 2017; Feyaerts et al., 2017, but also De Vries et al., 2021 specifically on ironic stance). These bodily resources include different elements, such as head tilts and shakes (Jehoul et al., 2017), nods (Mondada, 2009), shrugs (Jehoul et al., 2017), raised eyebrows (Feyaerts et al., 2022), frowning (Li, 2021), eye-gaze (Brône et al., 2017; De Vries et al., 2021 or De Vries et al., 2024), but also hand gestures (see Mondada, 2009; Jehoul et al., 2017). They all have different functions in interactions (for an overview, see Andries et al., 2023), ranging from an emotional perspective (such as surprise; see Ekman and Wallace, 2003) to negative/positive stance.

Some of these elements have been linked to humor production: head movements (Tabacaru, 2019 or De Vries et al., 2021), raised eyebrows and frowning (Tabacaru and Lemmens, 2014). Other non-verbal elements used with humor can include prosodic markers (Bertrand and Priego-Valverde, 2011; Attardo et al., 2013), but also smiling (Gironzetti, 2017) or laughter (Attardo, 2003).

The question is, then, to see the role these elements play with humor. In corpus studies conducted on non-spontaneous uses of humor, Tabacaru and Lemmens (2014) and, later, Tabacaru (2019) explain the role of *gestural triggers* for the shift from a serious meaning to a humorous/pretense meaning. In these contexts, speakers used raised eyebrows with salient elements for the humorous interpretation. The experiment was repeated with more spontaneous uses of humor for the French political debates prior to the presidential elections of 2017 (Tabacaru, 2020). The study shows that even in these types of interactions, which do not aim at being humorous (as opposed to television series and shows, stand-up comedy, etc.), speakers will make use of non-verbal elements in order to make their humorous message understood. In a study investigating eye-gaze in spontaneous interactions, De Vries et al. (2024) found that, in internal teases, the target of the teasing was focused on both verbally and visually, which then highlights the role these semiotic resources have in such settings.

Furthermore, Gironzetti (2022: 15), talks about different types of discourse markers that are used to signal “the speaker’s intentions by conveying a metamessage about how a certain utterance should be interpreted.” Or, as noted by Verhagen (2005: 22):

Linguistic expressions are primarily cues for making inferences, and understanding does not primarily consist in decoding the precise content of the expressions, but in making inferences that lead to adequate next (cognitive, conversational, behavioral) moves.

We can consider that speakers make assumptions and draw conclusions from their interlocutors’ (both verbal and non-verbal) behavior as discourse unfolds. As such, research should focus more on the multimodal side of discourse (Norris, 2004), as interactions include elements that are essential to the understanding of the (humorous) message. Speakers have to interpret their interlocutors’ non-verbal behavior, be it a gaze, facial expressions, body posture, etc., and respond to it accordingly.

As Stubbs (1986: 1; quoted in Englebretson, 2007:70) points out:

whenever speakers (or writers) say anything, they encode their point of view towards it … The expression of such speakers’ attitudes is pervasive in all uses of language. All sentences encode such a point of view, … and the description of the markers of such points of view and their meanings should therefore be a central topic for linguistics.

Englebretson (2007: 70) further notes that “every utterance enacts a stance,” hence, for the purpose of the present paper, the focus will fall on the humorous stance that is achieved in the corpus, which will be presented in the section below. As noted by De Vries et al. (2021: 37), it would make sense that such resources are used at specific times if they signal ironic intent. This is also the point made by Tabacaru (2019) where humor was made prominent through some sort of non-verbal element used by the speaker.

## Corpus and method

The examples presented here come from two American presidential debates: the first presidential debate between Donald Trump and Hillary Clinton in 2016 (@NBC News on YouTube), of a duration of 1h35m, and the first presidential debate between Donald Trump and Joe Biden in 2020 (©The Telegraph on YouTube), of a duration of 1h39m. Transcripts available online^2^ for the debates were also used, which consisted of more than 37.000 words. The settings of such debates are not as rich in humorous exchanges as television series (Brône, 2008, for example) or stand-up comedy shows, which are meant to be humorous by nature. But, as the debates present opposing candidates who need to surpass their adversary’s speech and persuade people to vote for them, humor will be used; such interactions also provide non-staged uses of humor, as opposed to other types of discourses mentioned before. They are also rich in interactional cues provided by the speakers. Hence, other meanings can emerge, because at the core of the debate is the wish to persuade people to vote for a certain candidate (Cobb and Kuklinski, 1997). For both these debates, the audience is reminded (either before or throughout the debate) that they are expected to remain silent, but there are several interruptions and moments when the audience/interlocutors laugh throughout the debates, especially in the first one, between Donald Trump and Hillary Clinton. Consequently, laughter cannot be considered a marker of humorous intent here (see also Hay, 2001),^3^ since the audience is not supposed to react to what is being said, which means that it is likely many humorous instances get no reaction from the public Nonetheless, it will be mentioned when the audience or the interlocutors laugh as a reaction to what is being said, because it marks an element of surprise and a clash between different layers of meaning.

The first debate contains 49 reactions that can be considered as including humorous intent, whereas the second debate includes 78 such reactions.^4^ Interestingly, as will be shown in the Discussion part, the first debate includes more facial expressions used with these humorous exchanges than the second one (63.7% of the corpus), although the second debate includes more examples.

The humorous examples have been annotated using ELAN^5^ regarding the facial/head markers used by the speakers when uttering them. Following the elements mentioned above, the attention falls on eyebrow movement (raised eyebrows and frowning), head movement (nods, shakes, tilts), shrugs and smiles. Hand movements were also used by the speakers, but they were not annotated in the corpus. When the face of the speaker/s was not visible during the humorous instances, it was marked as *not visible* in the corpus. This was mainly because of how the interlocutors were filmed during the debates, since the techniques are not consistent between the 2016 and the 2020 debate.

In the following section, examples from the corpus are presented to show how speakers make use of humor in order to build layers (Clark, 1996) of meaning and switch the viewpoint and the course of the conversation.

## Examples: discourse markers

Several instances are presented here, with the use of non-verbal elements that have been discussed in relation to humor (see Tabacaru, 2019 or De Vries et al., 2024). These markers include raised eyebrows, nods, shrugs and smiles.

In the example below, Trump is being sarcastic toward some of Clinton’s campaign strategies, such as the information available on her website, which she refers to for fact-checking purposes regarding his own claims. She refers to her website, which is followed by Trump’s interruptions. The key elements are the words “her website” which allow a shift of focus from a meaning that was intended as [good] to something that is [bad] (similar to example [1] above where the negative evaluation is added afterwards). For the sake of relevance, some of the lines have been removed from the example. The humorous instance is marked in bold with the facial expressions in red below. The facial expressions in bold show that the non-verbal markers happen simultaneously:

**Table:** 

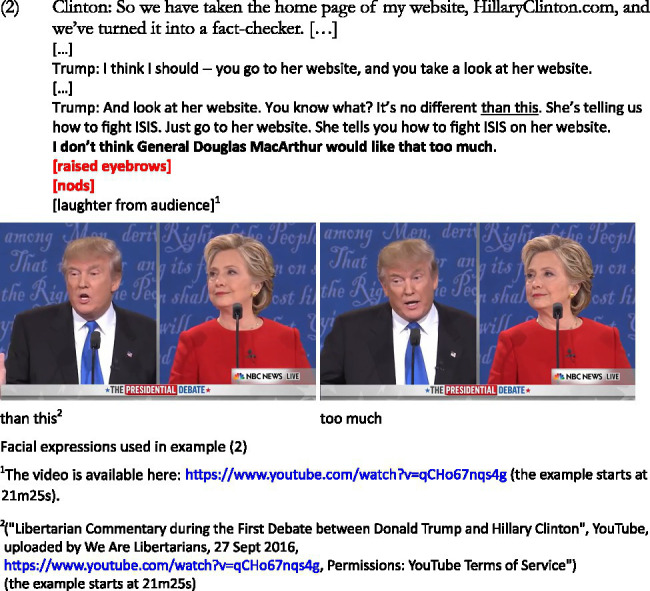

The sarcasm here is emphasized by Trump’s critical words and behavior (facial expressions, nods) used to talk about the type of information provided by Clinton’s website. The raised eyebrows used by Trump start when uttering *She tells you how to fight ISIS […]*,^6^ which makes Clinton the target of his sarcasm (see also Tobin and Israel, 2012 or Tobin, 2020). He raises his eyebrows and nods repetitively when uttering “I do not think General Douglas MacArthur would like that too much.” The mention of General Douglas MacArthur, military leader involved in both World War I and World War II, implies (Grice, 1975) that the strategies used by countries/leaders against their enemies should not be freely available online. The focus of attention is reversed: if, in Clinton’s words, her website provides useful information regarding fact checking, Trump sarcastically refers to the other information that it provides, such as strategies regarding the war on terror. The reference to General Douglas MacArthur metonymically^7^ gives access to the frame of successful military strategy, as opposed to Hillary Clinton’s position.

Clinton also uses Trump’s implied meaning in order to reverse the focus of attention once more, this time in her favor, but her facial expressions are different. Similar to findings by Gironzetti (2017) and Feyaerts et al. (2022), she uses a smile when implying that Trump does not have a plan regarding the war on terror, whereas she does:

**Table:** 

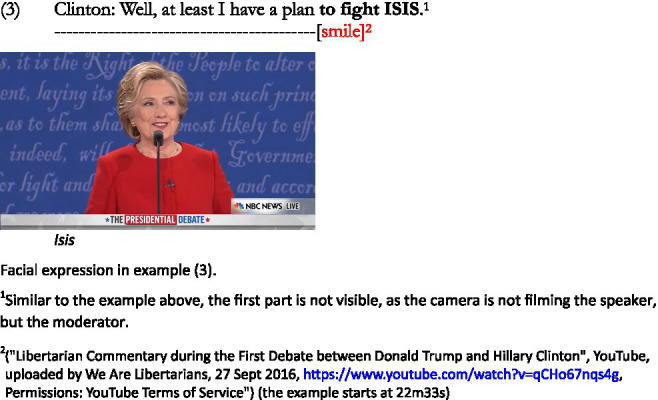

This exchange continues, as both speakers turn the tables against each other using elements given by their opponent. In the following, Trump explicitly says that making the information available online is counterproductive because military strategies should not be accessed by the enemy, thus making explicit what he implicitly stated at the beginning of this exchange:

**Table:** 

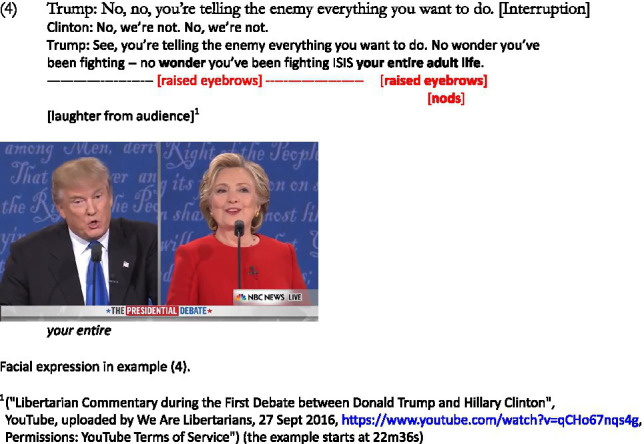

Trump here comes back to the information available on Clinton’s website, further focusing on the fact that the enemy would have access to her military strategy. His last line emphasizes that Clinton is unsuccessfully fighting ISIS because she has made her plans public this entire time. The two speakers thus have different viewpoints regarding the war on terror, Clinton trying to focus on what is good about her website (the fact-checking option), while Trump emphasizes on what is bad, giving rise to different sarcastic instances from both speakers, as can be summarized below:

**Table:** 

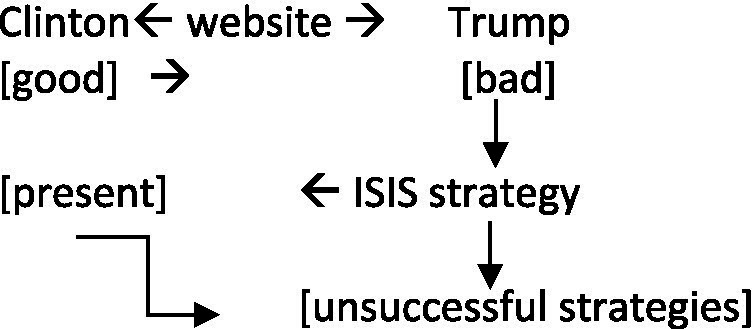

Both speakers here focus on what is beneficial to them, and it plays on different elements introduced by their interlocutor. The website is introduced by Clinton (see example [2] above) with a specific goal in mind, i.e., in order to propose a fact-checking option to verify the claims made, but Trump shifts the focus to the other information available there, such as military strategy, in order to show the negative side of the website. When Clinton picks up on this and defends her position by implying that, compared to him, she has already given this matter some thought, he further emphasizes the same idea, that she has been fighting the enemy unsuccessfully because they are already aware of her actions against them. Both interlocutors here enrich the common ground by defending their position and political actions (the case of Clinton) or by attacking their interlocutor (the case of Trump).

Raised eyebrows and nods are used in examples (2) and (4) with the sarcastic intentions of the speaker: when comparing Clinton’s military strategy to that of General Douglas MacArthur, and when introducing the comment on [her] entire adult life, which is again a case of generalization (similar to example [1] above). The marker used by Clinton is different, as she uses a smile when aligning with what her opponent said, which also creates a pretense space in which Trump does not have any plan on the war on terror, whereas she does. The personal pronouns *she* and *I* are used by the two participants, the former acting as direct criticism and the latter adding a positive spin on oneself while indirectly criticizing the opponent (as a third-person reference).

Another similar example is (5) below, where comparisons between the two positions are presented. Here, Trump is remined by the moderator that he claimed Mrs. Clinton does not have a “presidential look,” which he further argues using the word *stamina*. This word will be used by Clinton in her reply which emphasizes her experience in politics:

**Table:** 

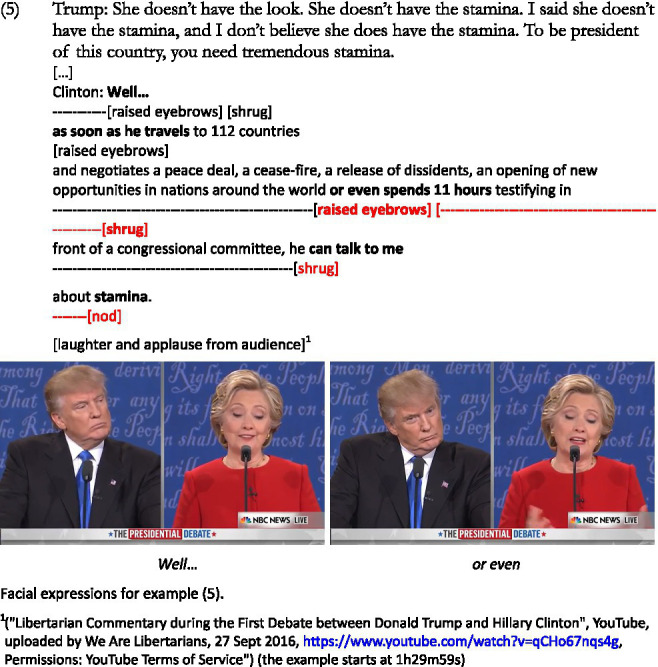

When starting her answer to Trump’s claim, Clinton uses a smile and raised eyebrows, as shown in above figure, which marks the start of her sarcastic response toward her opponent. This is also made manifest with the use of raised eyebrows again when uttering *or even* used to introduce other examples of her political experience. Other non-verbal elements include shrugs (after *Well*, with *or even* and *he can talk*), and nods when uttering the word *stamina*, which Trump had used.

Trump uses the word *stamina* four times to make a comment on his opponent’s potential competence as future president of the United States, to which Clinton answers using examples of her experience in politics, of which her opponent has none (the personal pronouns *she* and *he* are used by the two speakers to refer to each other directly). This, again, has to be interpreted against a shared common ground: Trump is not a politician, whereas she is. Her entire reply can be considered sarcastic as it represents a comparison to the absence of experience from Trump, but she uses certain discourse markers when she introduces her reply, in the middle of her argument to emphasize her claims, and again when uttering the word *stamina*, at the end.

Another example comes from the second debate, where, comparable to the repetition of the word *stamina* in the example above, Joe Biden repeats the word *segment*, used by the moderator, but adds a sarcastic meaning to it:

**Table:** 

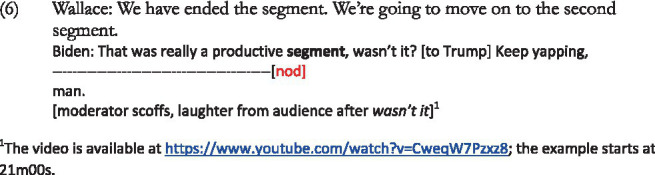

The word *segment* is uttered with a simple nod, which marks the repetition of the word used by the moderator, who had to intervene several times because of the interruptions between the speakers. Biden’s implied meaning here is that they could not speak freely, without interruptions, which is further emphasized by his *Keep yapping, man*, aimed at his political adversary. The element of incongruity is very salient here, as Biden means the opposite of what he says: the segment that just finished was not productive as one of the speakers kept interrupting the other. Here, the participants and the audience have access to the same common ground that was created during the debate: the speakers kept talking at the same time, which means that their positions could not be successfully presented. Interestingly, the nod comes with the word *segment*, and not the adjective *productive*, which adds the pretense layer in Biden’s remark. It is comparable to the example above where Clinton also nods when uttering stamina, the word previously used by her opponent. These represent key elements (Brône, 2008) that mark the switch between the two layers of meaning.

A final example with such repetitions is (7) below, in which the topic of taxes and jobs is discussed:

**Table:** 

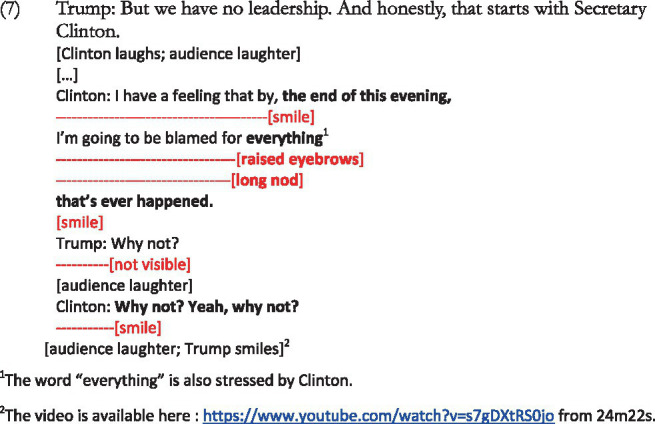

Another case of generalization is the word *everything* sarcastically uttered by Clinton with marked non-verbal elements such as raised eyebrows, head movement, and a prosodic stress in order to accentuate that she is being accused of different things throughout the debate, such as is the case here. This follows Trump’s position that he is not a politician, whereas she is (me versus them), not only a Democrat, but also Secretary of State, so, in a position of power and can metonymically refer to the frame of power. The key element *everything* marks sarcasm and exaggeration (Tabacaru, 2019), which is targeting her opponent, who keeps blaming her for different issues. This is very similar to examples (2) and (3) above, where Trump names her directly (through personal pronouns in the previous examples), whereas Clinton responds using the first person: *I have a feeling […] I’m going to be blamed*. If the focus of attention falls on her through her opponent’s words, she shifts it through the use of exaggeration. The answer given by Trump, humorous as well, adds a layer not to the exaggeration to which she points, but to the word *blame*, with the rhetorical question meaning: *why should she not be blamed for everything*? Clinton then echoes Trump’s words *why not* twice at the end, which are uttered with a smile, followed by a smile from Trump as well. All these lines represent sarcasm/humor, targeting the adversary, and all of them include non-verbal elements (except the one that is not visible, uttered by Trump).

## Results

The results for the annotations regarding semiotic markers in the two debates considered here can be seen in Table 1. Unfortunately, in 13% of cases, the face of the speaker/s could not be seen (they were either not at all filmed or filmed from a distance, in which case the facial expressions could not be identified). There is also a clearer view in the first debate (filmed from the front) as opposed to the second debate, which included more examples of (humorous) interruptions, where the face of the speaker could not be seen. Similarly, the first debate between Trump and Clinton includes more non-verbal elements used with humor than the second one, although the number of humorous interactions was higher in the second debate compared to the first one.

**Table 1:** Results of facial expressions in the two debates.

	Trump vs. Clinton	Trump vs. Biden	Total
Raised eyebrows	52	19	71
Nod/s	43	5	48
Not visible	3	25	28
Frown	7	8	15
Head shake/s	9	5	14
Head tilt	4	9	13
Shrug	7	4	11
Smile	10	1	11
Raised eyebrow	2	2	4
Total	137	78	215

**Figure 1:**
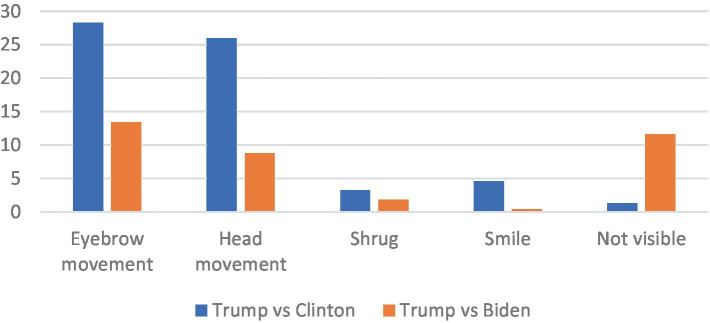
Results for discourse markers in the two debates.

For a clearer view of the discourse markers used in these humorous exchanges, the different eyebrows movements, head movements, and other markers have been considered together, as is shown in Figure 1. All eyebrow movements (raised eyebrows together, raising one eyebrow or frowning) and head movements (head tilts, shakes, or nods) are considered as two categories, for a general view of such resources.

These results show a preference for eyebrow and head movement, similar to other studies conducted on staged interactions (Tabacaru and Lemmens, 2014; Tabacaru, 2019), but also on spontaneous interactions (for example, De Vries et al., 2024). In 41.8% of cases of humor, speakers either used raised eyebrows or a frown, as shown in the examples presented above. The same goes for head movements, which are used in 34.8% of the data. The figure above also shows different behavior patterns for the two debates: both eyebrows and head movement are used more in the first debate (2016) than in the second one (2020), in which there were also more cases of markers that could not be analyzed for lack of visibility in the given videos (11.6% compared to 1.3%). Smiles are also used by the speaker/s, specifically in the first debate. This iscomparable to results presented by Gironzetti (2024), for example, although the intensity of such smiles has not been studied here, as has been the case elsewhere (see Gironzetti et al., 2016 or Ergül et al., 2024).

Of course, these markers are not specific to humor and are used in discourse in general (see also Tabacaru, 2019). For example, Jehoul et al. (2017) report that a shrug marks obviousness, which can also be the case here, but from a pretense space perspective. If we take example (5), Clinton shrugs several times during her answer to Trump’s attacks: the first one can be considered a marker of obviousness, especially since it is used with “Well,” but from a sarcastic perspective, i.e., it is obvious that Trump does not have her political experience. The shrug used with “he can talk to me [about stamina]” marks a pretense space, as it is implied that he will never have as much experience as she does. Debras (2013) mentions the shrug as marker of disengagement and this could also apply here, as Clinton seems to adopt an indifferent attitude toward Trump’s attacks given her political experience (i.e., me versus him, as she also refers to him in the third person).

The examples presented here represent examples of sarcasm (which is considered a type of humor; see Tabacaru, 2019 or Tabacaru, 2020), where the target of the ‘attack’ is clear (Attardo, 2000, for example). The sarcastic meaning/implicature has then to be made clear with the use of both verbal and non-verbal markers.

## Conclusion

This paper has explored the question of humorous stance in American political settings, of which two debates were presented in order to analyze how non-verbal markers were used by the speakers to make their meanings understood. Interactions such as these provide richer contexts for discourse analysis, although humor is not necessarily encouraged (this is the case for both debates analyzed here), as opposed to spontaneous interactions of humor between friends, for example. Nevertheless, the data shows the pervasiveness of humor, which can be used as a political tool in such contexts, as has been shown elsewhere (Li and Pryor, 2020, for instance). Humor is a mechanism that “appears frequently in interaction” (Priego-Valverde, 2024: 1), which seem to be case here as well. In a setting in which participants are not expected to ‘amuse’ the audience, humor still happens as a way to shift the focus of attention (for example, from a [good] scenario to a [bad] scenario like is the case with the Covid-19 regulations) or to counter the adversary’s attacks (as is the case with Clinton’s focus on her political experience). As such, it is an interesting area of research in political settings.

The purpose of the paper was to show the way non-verbal discourse markers are used in interactions in order to create humorous meanings, and new layers built on the common ground (known or enriched during the debates). Such layers introduce pretense spaces of meaning aimed at the political opponent the speaker is debating. They also contribute to the topic of stance, as they show divergent viewpoints toward the same object/topic. In the case of political debates, humorous stance can thus play a role in the issue of persuasion because it emphasizes the interlocutors’ ability to “turn the tables” on their opponent (Brône, 2008).

Although the videos used did not provide absolute view on the faces of the speakers, they contribute to showing the use of such markers with humorous intent. Similar to a key element in the case of hyper-understanding and misunderstanding (Brône, 2008), these non-verbal markers allow an emphasis on parts of speech that are relevant for a humorous interpretation. The results show a preference for both facial expressions and head movements, similar to staged interactions. Although the data is not balanced, neither in the way the speakers were filmed nor in the way they use these markers throughout the debates, they do provide a more detailed understanding on the way humor is a tool of human communication that is used for different purposes.

## Data Availability

The raw data supporting the conclusions of this article will be made available by the authors, without undue reservation.

## Footnotes

^1^This also echoes Langacker’s (1987) construal relationship and figure-ground reversal.

^2^The commission on presidential debates: https://www.debates.org/voter-education/debate-transcripts/.

^3^De Vries et al. (2021) found that laughter happens more in ironic than non-ironic instances.

^4^The identification of these instances was done using the theories and methods presented in Feyaerts (2013b) and Tabacaru, 2019. For example, Tabacaru (2019, p. 98) carried out reliability tests on humorous and non-humorous instances, with 84% agreement between the corpus and the participants to the reliability tests. It is safe to assume that humorous instances can be identified in either a written or oral corpus without the help of laughter.

^5^ELAN is a tool for video annotation freely available for researchers from the Max Planck Institute for Psycholinguistics, The Language Archive, Nijmegen, The Netherlands (http://tla.mpi.nl/tools/tla-tools/elan/); see Brugman and Russel (2004), Wittenburg et al. (2006), and Sloetjes and Wittenburg (2008). I have used version 6.4.

^6^The raised eyebrows are not visible at all times as the camera zooms out, but they are visible in the beginning and the end of his reaction. The screenshot here includes the very visible part at the end.

^7^See, for example, Feyaerts (1999) or Tabacaru and Feyaerts (2016).
